# Free and Poly-Methyl-Methacrylate-Bounded BODIPYs: Photodynamic and Antimigratory Effects in 2D and 3D Cancer Models

**DOI:** 10.3390/cancers15010092

**Published:** 2022-12-23

**Authors:** Marco Ballestri, Emanuela Marras, Enrico Caruso, Fabrizio Bolognese, Miryam Chiara Malacarne, Elisa Martella, Matilde Tubertini, Marzia Bruna Gariboldi, Greta Varchi

**Affiliations:** 1 Institute for the Organic Synthesis and Photoreactivity, Italian National Research Council, 40129 Bologna, Italy; 2 Department of Biotechnology and Life Sciences (DBSV), University of Insubria, 21100 Varese, Italy

**Keywords:** photodynamic therapy, BODIPYs, poly-methyl methacrylate nanoparticles, electrostatic loading, inhibition of cells migration, antitumor efficacy, drug delivery

## Abstract

**Simple Summary:**

Photodynamic therapy (PDT) is a minimally invasive and highly selective technique to treat solid tumors and other malignancies. To exert a significant cytotoxic effect, PDT must simultaneously gather a photosensitizer (PS), a light at a specific wavelength, and oxygen. Although several PSs have been developed so far, systems with higher selectivity and efficacy are still needed to improve PDT anticancer treatment. This work shows how BODIPYs photosensitizers, loaded onto polymethyl methacrylate nanoparticles, can effectively reduce tumor cell viability in vitro and lower their migratory ability, thus, potentially reducing the metastatic tumor potential.

**Abstract:**

Several limitations, including dark toxicity, reduced tumor tissue selectivity, low photostability and poor biocompatibility hamper the clinical use of Photodynamic therapy (PDT) in cancer treatment. To overcome these limitations, new PSs have been synthetized, and often combined with drug delivery systems, to improve selectivity and reduce toxicity. In this context, BODIPYs (4,4-difluoro-4-bora-3a,4a-diaza-s-indacene) have recently emerged as promising and easy-to-handle scaffolds for the preparation of effective PDT antitumor agents. In this study, the anticancer photodynamic effect of newly prepared negatively charged polymethyl methacrylate (nPMMA)-bounded BODIPYs (**3@nPMMA** and **6@nPMMA**) was evaluated on a panel of 2D- and 3D-cultured cancer cell lines and compared with free BODIPYs. In particular, the effect on cell viability was evaluated, along with their ability to accumulate into the cells, induce apoptotic and/or necrotic cell death, and inhibit cellular migration. Our results indicated that **3@nPMMA** and **6@nPMMA** reduce cancer cell viability in 3D models of HC116 and MCF7 cells more effectively than the corresponding free compounds. Importantly, we demonstrated that MDA-MB231 and SKOV3 cell migration ability was significantly impaired by the PDT treatment mediated by **3@nPMMA** and **6@nPMMA** nanoparticles, likely indicating the capability of this approach to reduce metastatic tumor potential.

## 1. Introduction

Photodynamic therapy (PDT) is a therapeutic modality applied to the treatment of several diseases, such as acne, infections, and cancer [1]. In PDT, cytotoxic reactive oxygen species (ROS), mainly singlet oxygen (^1^O_2_), are generated locally by the combined action of a photosensitizer (PS) and light, and selectively eradicate diseased cells. One of the main advantages of PDT relies on its potential dual selectivity, e.g., the preferential PS accumulation at the diseased tissue and the focused light irradiation of the target area, making it a minimally invasive therapeutic option, especially for anticancer treatment.

The preferential accumulation of the PS at the target site is a crucial condition to improve PDT selectivity and efficacy and to reduce possible side effects in healthy tissues [2,3,4,5]. Several PSs have been described so far, and some have been proposed, or marketed, for clinical use [5]. However, to overcome the limitations of the current PSs, and to widen their application and favor a more effective translation of PDT to clinics, it is necessary to develop more selective and efficient PSs [2].

The most critical limitations of current PSs include dark toxicity, poor tumor tissue selectivity, low molar extinction coefficients, particularly in the therapeutic spectral window (e.g., 650–875 nm), low photostability and poor biocompatibility. In addition, the synthesis, chemical modification, and purification of the most clinically relevant PSs, such as cyclic tetrapyrroles (porphyrins, chlorins, and bacteriochlorins), are complex and low yielding procedures. Therefore, there is a call for the development of novel PS classes [6].

In this context, the use of BODIPYs (4,4-difluoro-4-bora3a,4a-diaza-s-indacene) as scaffolds to develop PSs with improved features for anticancer applications has recently emerged as a promising option [6,7,8,9].

Due to their outstanding chemical and physical features, such as high molar extinction coefficients in the visible region, high resistance to photobleaching, and extreme stability under different environmental conditions, BODIPYs were first developed for various photonics applications [10,11,12,13]. Furthermore, the high chemical versatility of the BODIPY’s core allows for fine modulation of different key photophysical features [5,6,14]. For instance, although BODIPY dyes usually exhibit high fluorescence quantum yields, which is incompatible with their use in PDT, fluorescent BODIPYs can be easily chemically modified, such as through di-iodination of the boron–dipyrrolylmethene core [15], enabling them to generate ROS under light irradiation, and, thereby, transforming them into efficient PSs [5,16].

Interestingly, the chemical modulation of the BODIPY scaffold may lead to a balanced ^1^O_2_ photo-generation/fluorescence ratio, which is an interesting property for developing PDT/bioimaging theragnostic agents [17].

The combination of PSs with drug-carrier nanoparticles, i.e., gold, silica, and polymeric, is a successful approach to improve solubility, selective targeting, and delivery of PSs for PDT applications, leading to better control of the transportation of the PS and its selective accumulation in tumor tissues [18]. Moreover, the application of nanoparticles in cancer therapy and diagnosis has other potential advantages, including overcoming multidrug resistance and preventing enzymatic degradation of the active agent [19].

During the past decade, we have reported the use of positively charged, core-shell poly-methyl methacrylate nanoparticles (PMMA@NPs) electrostatically loaded with negatively charged PSs, e.g., porphyrins and phthalocyanines, for in vitro and in vivo anticancer photo- and sono-dynamic applications [20,21,22,23]. One possible drawback of this system lies in the high positive charge of the NPs, which could reduce their biocompatibility, and increase intrinsic toxicity. As recently reviewed, positively charged and hydrophobic NPs are more prone to interact with the cell membrane, be internalized, and induce oxidative stress, autophagy, and apoptosis [24]; therefore, inducing higher carrier-related toxicity.

To avoid these potential side effects, we herein report, for the first time, the synthesis of negatively charged core–shell PMMA nanoparticles (nPMMA), electrostatically loaded with two positively charged, di-iodinated BODIPYs [25]. The presence of the iodine atoms on the BODIPYs’ core induces the well-known intersystem crossing effect, causing a nearly complete inhibition of fluorescence in favor of a very high rate of singlet-oxygen generation [15]. Therefore, to extend the scope of our study, the corresponding non-iodinated and fluorescent analogs of BODIPYs were synthesized and exploited for performance in vitro cellular internalization studies.

Extensive physical–chemical characterization allowed establishment of their size, shape, zeta potential, loading and release abilities. The anticancer photodynamic effect of these newly prepared nPMMA-bounded BODIPYs was evaluated on a panel of cancer cell lines, e.g., colon cancer cells (HCT116), ovarian cancer cells (SKOV3), and breast cancer cells (MCF7 and MDA-MB231) and compared with free BODIPYs. In particular, the effect on cell viability was evaluated, along with their ability to accumulate into the cells, induce apoptotic and/or necrotic cell death, and inhibit cellular migration. To achieve more insight into the potential of these novel nanosystems as PDT anticancer agents, we evaluated their performance in HCT116 and MCF7 cells grown as 3D spheroids. Compared to monolayer cell lines, the 3D model better recapitulates the in vivo avascular tumor characteristics, such as normoxic/hypoxic regions and cell–cell interactions, thus representing a more suitable benchmark for evaluating the PDT effect [26]. Thus, the effects on cell growth and cell viability and the ability of free and nPMMA-bounded BODIPYs to penetrate through extracellular spaces in the spheroids and accumulate in the cells were assessed.

## 2. Materials and Methods

### 2.1. Reagents and Chemicals

The 3-Sulfopropyl methacrylate potassium salt, methyl methacrylate (MMA), sodium dodecyl sulphate, potassium persulphate (KPS) and all chemicals required for preparation and characterization of the nanoparticles were purchased from Aldrich (Milan, Italy) and used without further purification. All reagents for cell culture and in vitro experiments were purchased from Euroclone (Milan, Italy).

### 2.2. BODIPYs Synthesis

Non fluorescent BODIPYs, **3** and **6,** were synthesized, as previously described [25]. The corresponding fluorescent analogs, **3f** and **6f,** were obtained as follows: dry pyridine (8 mL/mmol) was added to derivative **1a** (or **1b**, 1 eq., Figure S1) [27] under an argon atmosphere, at room temperature. After 6 h, additional pyridine (4 mL/mmol) were added to the reaction mixture. After stirring at room temperature for 24 h, dry ethyl ether was added, and the upper layer (unreacted starting material) removed. The sticky residue was then dissolved in water and washed several times (3–4) with distilled ethyl ether to remove the residual starting material and pyridine. The aqueous phase was then freeze dried to afford derivatives **3f** and **6f** as pure compounds, in 82% and 56% yield, respectively.

Figures S2 and S3 show the 4,4-difluoro-1,3,5,7-tetramethyl-8-(4-(4-bromopyridiniobuthoxy)phenyl)-4-bora-3a,4a-diaza-s-indacene (**3f**).

^1^H NMR (400 MHz, DMSO-d6) *δ* 9.13 (d, *J* = 5.8 Hz, 2H), 8.62 ((t, *J* = 6.2 Hz, 8.18 (t, *J* = 6.2 Hz, 2H), 7.26 (d, *J* = 8.5 Hz, 2H), 7.09 (d, *J* = 8.6 Hz, 2H), 6.17 (s, 2H), 4.70 (t, *J* = 7.4 Hz, 2H), 4.07 (t, *J* = 6.2 Hz, 2H), 2.44 (s, 6H), 2.14–2.11 (m, 2H), 1.79–1.76 (m, 2H), 1.38 (s, 6H). ^13^C NMR (101 MHz, DMSO-d6) *δ* 159.53, 146, 02, 145.27, 142.54, 129.62, 128.60, 126, 50, 121,74, 115.57, 67.42, 60.97, 55.35, 28.14, 14.61.

Figures S4 and S5 show the 4,4-difluoro-1,3,5,7-tetramethyl-8-(4-(4-bromopyridiniooctanoxy)phenyl)-4-bora-3a,4a-diaza-s-indacene (**6f**).

^1^H NMR (400 MHz, DMSO-d6) *δ* 9.09 (d, *J* = 5.9 Hz, 2H), 8.59 (t, *J* = 7.8 Hz, 1H), 8.15 (t, *J* = 6.9 Hz, 2H), 7.22 (d, *J* = 8.4 Hz, 2H), 7.14–7.01 (m, 2H), 6.15 (s, 2H), 4.59 (t, *J* = 7.5 Hz, 2H), 4.00 (t, *J* = 6.4 Hz, 2H), 2.42 (s, 6H), 1.91 (m, 2H), 1.71 (p, *J* = 6.8 Hz, 2H), 1.44–1.17 (m, 14H). ^13^C NMR (101 MHz, DMSO-d6) δ, 159.73, 155.10, 145.94, 145.21, 143.14, 131.60, 129.56, 128.56, 126.27, 121.72, 115.57, 68.04, 61.22, 31.13, 29.03, 28, 99, 28.76, 25.86, 25, 79, 14, 59.

### 2.3. BODIPYs Characterization

BODIPYs 1-octanol-water partition coefficient (P) was determined at 25 °C by means of equal volumes of pre-equilibrated milliQ water (3 mL) and 1-octanol (3 mL). Specifically, an aqueous solution of the BODIPYs (40 µM) was stirred for 8 h at 25 °C in the presence of octanol, then, 200 µL of both aqueous and organic phases were diluted with DMF up to 2 mL and the BODIPY final concentration was determined by absorption spectroscopy (Cary 50, Agilent Technologies, Milan, Italy). The relative rates of ^1^O_2_ produced by the BODIPYs were experimentally measured by monitoring the disappearance of the 410 nm absorbance band of 1,3-diphenylisobenzofuran in isopropanol, an ^1^O_2_ scavenger, and normalized with respect to values obtained using Rose Bengal as standard [5].

### 2.4. Nanoparticles Synthesis and Characterization

An aqueous solution (100 mL) of 3-sulfopropyl methacrylate potassium salt (SPM, 0.65 mmol, 151 mg) and sodium dodecyl sulfate (SDS, 0.01 mmol, 3 mg) was put into a 250 mL three-neck reactor under mechanical stirring and bubbled with a nitrogen flux. After 15 min, methyl methacrylate (MMA, 4 mL, 40.2 mmol) was added dropwise and heated at 80 °C. Once the temperature reached 80 °C the radical initiator, potassium per-sulfate (KPS, 0.25 mmol, 93 mg), was introduced into the solution. The resulting white suspension was left under heating and stirring for 4 h and then cooled at room temperature. After cooling, the milky suspension was dialyzed for 5 days, changing the outer water twice a day (dialysis Visking tubing; Φ = 28.6 mm; MWCO = 12–14 KDa).

The hydrodynamic diameter and zeta-potential were analyzed at 25 °C through dynamic light scattering (DLS) measurements on a NanoBrook Omni Particle Size Analyzer (Brookhaven Instruments Corporation, New York, NY, USA). The nanoparticles’ morphology was analyzed by scanning electron microscopy (SEM) on a Philips XL30, and the sample was prepared by drying a droplet of NP solution (0.5 mg/mL or 1 mg/mL).

#### 2.4.1. Nanoparticle Loading, Stability and Release Experiments

Briefly, positively charged BODIPYs were combined with nPMMA nanoparticles by electrostatic interaction: a certain amount of BODIPY (typically 500 μg) was dissolved in THF or acetone at a concentration of 0.5 mg/mL and added to an aqueous solution of negatively charged PMMA (5 mg/mL) in an Eppendorf vial, and then stirred in a Vortex apparatus for 20 s at room temperature. Each sample was then centrifuged for 30 min at 4 °C (48000 RCF) in an Allegra 64R centrifuge (Beckman Coulter, rotor F1202). Upon centrifugation the supernatant was removed and the remaining pellet was resuspended and washed with milliQ water till cleared of unbounded BODIPY, as determined by UV–Vis analysis (Cary 100, Agilent Technologies, Milan, Italy). To increase BODIPYs’ loading, this procedure was iteratively repeated three times.

The loading content was evaluated by recording the absorption spectra of the filtrates by UV–Visible spectroscopy and comparing to a calibration curve of free BODIPYs obtained by plotting the concentration against their absorbance at 530 nm.

The loading efficiency and content were then calculated, based on the following formula:

Loading efficiency (LE, %) = amount of BODIPY loaded onto NPs (μg)/amount of BODIPY initially used (μg) × 100

Loading content (LC, %) = amount (μg) of loaded BODIPY/amount (μg) of nanoparticles × 100

In vitro stability studies were performed over time (48 h) at 37 °C in FBS 20% in phosphate buffered saline (PBS; pH 7.4). All experiments were carried out in the dark to avoid possible disassembly contribution, due to ROS formation under natural light exposure. In a typical experiment, 10 μL of nanoparticle solution (NPs conc: 1 mg/mL; BODIPY conc: 15 μg/mL) were diluted with 1 mL of the medium, while being maintained at 37 °C. Changes in particle size distribution over time were monitored by means of DLS.

The corresponding **3f@nPMMA** and **6f@nPMMA** nanoparticles were obtained and characterized following the same procedures described for non-fluorescent derivatives **3** and **6**. Briefly, 20 μg of **3f** (or **6f**) was dissolved in water and loaded, as previously reported, onto nPMMA (1 mg/mL). This was followed by three cycles of centrifugation/washing to remove unloaded BODIPYs.

The release of BODIPYs from the nanoparticles was measured by spectrophotometric analysis on **3f@nPMMA** and **6f@nPMMA**. An amount of 1 mL of the obtained suspension was inserted into a dialysis tube (Φ = 1 cm; MWCO = 12–14 KDa) and placed into a sealed 20 mL plastic container. Then, 6 mL of PBS were added to the container, which was kept shaking at 37 °C for a total of 48 h. At selected time points, 1 mL of the external solution was withdrawn and replaced with fresh PBS, and the amount of fluorescent BODIPY was determined against its corresponding calibration curve, obtained by plotting the concentration from 1 μg/mL to 5 μg/mL against their respective absorbance at 596 nm.

#### 2.4.2. Reactive Oxygen Species (ROS) Determination

The Reactive Oxygen Species (ROS) production was evaluated for **3@nPMMA** and **6@nPMMA** using the chemical probe 2,7-dichlorodihydrofluorescein diacetate (H_2_DCFDA) that, in the presence of ROS, is converted to fluorescent 2,7-dichlorofluorescein (DCF). For the probe preparation, 2 mL of NaOH (0.01 M) were added to 500 µL of a methanol solution of H_2_DCFDA (1.1 mM) and stirred for 30 min at room temperature. Then, 10 mL of phosphate buffer (pH = 7.4) were added to obtain the final ROS probe solution. For ROS detection, samples were prepared as follows: a certain amount of **3@nPMMA** and **6@nPMMA** solutions, corresponding to a BODIPYs’ final concentration of 4 µg/mL was added to a cuvette containing 500 µL of phosphate buffer and 218 µL of ROS probe, and water was added to a final volume of 1 mL. Samples were then irradiated with a white 320–700 nm emission wavelength tungsten lamp (300 W, light intensity 45 mW/cm^2^ at 670 nm, Phillips, Bologna, Italy). The light was positioned at a distance of 40 cm from the cuvette, and the absorption spectra were recorded at different time points with a Cary 100 UV–Vis spectrophotometer (Agilent Technologies, Milan, Italy), reading absorbance at 500 nm.

### 2.5. In Vitro Biological Studies

#### 2.5.1. Cell Lines and In Vitro Culture Conditions

The human colorectal cancer HCT116, breast adenocarcinoma MCF7 and MDA-MB231 and ovarian cancer SKOV3 cell lines were obtained from ATCC (American Type Culture Collection, Manassas, VA, USA). Cells were maintained under standard culture conditions (37 °C; 5% CO_2_) in RPMI-1640 medium (MCF7, and MDA-MB231 cells) or in DMEM medium (SKOV3 and HCT116 cells) supplemented with 10% fetal calf serum, 1% glutamine and 1% antibiotics mixture. For HCT116 and SKOV3 cells, 1% sodium pyruvate and 1% non-essential amino acids were also added to the culture medium.

To produce the corresponding spheroids, HCT116 and MCF7 cells were grown as a monolayer, detached, and 2.5 × 10^3^ cells/well were then seeded onto 96U plates Nunclon Sphera (Thermo, Milan, Italy) and incubated at 37 °C in a 5% CO_2_ atmosphere. Spheroids were used at day 7 from seeding.

Unless otherwise indicated, cells were seeded and allowed to attach and growth for 48 h before treatment with the compounds. After 24 h of incubation at 37 °C, the drug-containing culture medium was removed, and cells were irradiated for 1 h in drug-free PBS and then incubated in the dark for 24 h before performing the assays. The irradiation step was always performed using a green LED lamp composed of 12 × 3 W diodes placed on a 11 cm diameter disk and equipped with a heat sinker and with a maximum emission at 525 nm and a width at half maximum of 70 nm (fluence rate 3.036 × 10^−3^ W/cm^2^).

#### 2.5.2. Cell Viability Assays on 2D and 3D Dimensional Models

Cell survival following exposure to the free- and nPMMA-bounded BODIPYs was evaluated using the MTT assay, as described elsewhere [28]. Cells were seeded onto 96-well plates (3 × 10^3^ cells/well) and treated with a range of drug concentrations (0.1–50 nM). At the end of the previously described experimental protocol (Section 2.5.1), MTT reagent was added to each well at a final concentration of 0.4 mg/mL for 3 h at 37 °C. Cell viability was determined by measuring the absorbance at 570 nm, using an iMark Microplate Reader (BIORAD), following dissolution of formazan crystals, formed by MTT metabolism in viable cells.

Possible intrinsic (i.e., not photodynamic) effects on cell viability, due to BODIPYs (free or nPMMA-loaded) without irradiation, were assessed by exposing cells to concentrations ten-fold higher than those used in the PDT experiments, but excluding the irradiation step. The IC_50_ values (i.e., the concentration affecting 50% of cell survival fraction) were obtained by nonlinear regression analysis using the GraphPad PRISM 4.03 software (GraphPad Software Inc., San Diego, CA, USA).

The phototoxic effects of **3**, **3@nPMMA**, **6** and **6@nPMMA** on HCT116 and MCF7 spheroids were assessed, based on a dye exclusion assay. Briefly, spheroids were treated with the compounds at concentrations corresponding to the IC_50_ values obtained by the MTT assays on HCT116 and MCF7 cells cultured as monolayers. After 24 h incubation, following replacement of the drugs-containing medium with PBS, spheroids were irradiated under a green LED light for 1 h (fluence rate 3.036 × 10^−3^ W/cm^2^). After irradiation, spheroids were incubated in the dark at 37 °C in a drug-free medium, and, after 24, 48 and 72 h, three to five spheroids for each treatment were independently collected, disaggregated using trypsin–EDTA solution, and live cells were counted using a Burker hemocytometer, following Trypan Blue staining. Control spheroids were treated only with culture medium and incubated/irradiated as were the treated ones.

#### 2.5.3. Flow Cytometric Analysis

The percentage of apoptotic and necrotic cells was evaluated by flow cytometric analysis. For this set of experiments, cells were exposed to the BODIPYs as free or loaded onto nPMMA at their respective IC_50_ values. To assess the percentage of apoptotic cells, at the end of treatment, both adherent and detached cells were harvested, washed in PBS, and fixed in 70% ethanol at −20 °C for 30 min. After a further wash in PBS, the DNA was stained with a solution of propidium iodide (PI) in PBS (50 μg/mL) in the presence of RNAse A (30 U/mL) at room temperature for 15 min before analyzing the samples. Intracellular accumulation of the BODIPYs was performed in all cell lines cultured as monolayer and exposed to the free and nPMMA-bounded fluorescent **3f** and **6f** (100 nM for 24 h). At the end of the exposure time, treated cells were detached by trypsinization, washed thoroughly in ice-cold PBS, resuspended in PBS and analyzed, exploiting the intrinsic fluorescence of these compounds. All samples were analyzed with a FACSCalibur flow cytometer (Becton Dickinson Mountain View, CA, USA) and data were processed using CellQuestPRO software (Becton Dickinson). Fluorescent emission of PI was collected through a 575 nm band-pass filter, acquired in log mode, and the percentage of apoptotic cells in each sample was determined, based on the sub-G1 peaks detected in mono-parametric histograms. BODIPY fluorescence was collected through a 530-nm band-pass filter and BODIPY accumulation was quantitated in arbitrary units, based on the median fluorescence intensity (MFI).

#### 2.5.4. Effects on Cell Migration

The effects on cell migration eventually induced by **3**, **3@nPMMA**, **6** and **6@nPMMA** were evaluated on SKOV3 and MDA-MB231 cell lines and assessed by the Scratch Wound Healing assay. Cells were seeded onto 6-well plates (MDA-MB231: 1.3 × 10^5^/well; SKOV3: 0.7 × 10^5^/well) and allowed to grow for 48 h (approximately to confluence) followed by a 24 h treatment with subtoxic concentrations, corresponding to their respective IC_20_, of **3** (0.5 nM), **3@nPMMA** (1 nM for MCF7; 0.5 nM others), **6** (0.2 nM for MDA-MB231; 0.4 nM others) and **6@nPMMA** (1 nM for HCT116; 1.5 nM for MDA-MB231; 2.5 nM others). A scratch was made with a pipette tip, and the drug-containing medium was replaced by fresh PBS, and cells were irradiated with a green LED light for 1 h (fluence rate 3.036 × 10^−3^ W/cm^2^) followed by incubation in a drug-free medium at 37 °C for 24 h. Pictures of the scratch wound were taken immediately after the irradiation step (t0) and after 24 h through a camera connected to an Olympus IX81 microscope.

#### 2.5.5. Western Blot Analysis

The expression of MMP2 and MMP9 in whole cell lysates following treatment with **3**, **3@nPMMA**, **6** and **6@nPMMA**, at concentrations corresponding to their respective IC_20_, was detected by Western blot analysis of whole cell lysates. Briefly, cells were resuspended in lysis buffer (NaCl 120 mM, NaF 25 mM, EDTA 5 mM, EGTA 6 mM, sodium pyrophosphate 25 mM in tris-buffered saline TBS 20 mM pH 7.4, phenylmethanesulfonyl fluoride PMSF 2 mM, Na_3_VO_4_ 1 mM, phenylarsine oxide 1 mM, 1% NP-40 and 10% Protease Inhibitor Cocktail), incubated for 10 min on ice after adding SDS (final concentration 0.1%) and lysates were collected by centrifugation (12,800 rpm for 20 min). Protein concentration was determined by the BCA assay (Pierce, Italy) and 50 μg of protein per sample were loaded onto 8% polyacrylamide gels and separated under denaturing conditions. Protein bands were then transferred onto Hybond-P membranes (Amersham Biosciences, Italy) and Western blot analysis was performed by standard techniques with mouse monoclonal antibody directed against MMP2 and MMP9 (Santa Cruz Biotechnology, Inc.). Equal loading of the samples was verified by re-probing the blots with a mouse monoclonal anti-actin antibody (Santa Cruz Biotechnology, Inc.). Protein bands were visualized through the G-box (Syngene, Chemi-XT4) using peroxidase-conjugated anti-mouse secondary antibodies (Sigma-Aldrich) and the Westar Supernova Substrate (Cyanagen). Densitometric analysis was performed by Image-J software.

#### 2.5.6. Localization and Diffusion of the PSs Inside Spheroids

Spheroids were obtained as reported at Section 2.5.1 and treated with the fluorescent version of the free- and nPMMA-bounded BODIPYs, **3f**, **6f**, **3f@nPMMA** and **6f@nPMMA**, at 100 nM concentration in the dark. After 24 h of incubation, PSs fluorescence intensity and distribution into spheroids were evaluated by confocal microscopy. Spheroids were transferred from 96-well plates to 35 mm dishes, washed with PBS and directly observed with a Leica SP5 Confocal Microscope. Images from poles to equatorial plane of the spheroids were acquired. The intensity of fluorescence in the spheroids exposed to the different BODIPYs were compared using LAS LAF software. No evidence of a different diffusion rate of the BODIPYs was seen through the acquired sections, so, therefore, the diffusion rate was analyzed exclusively at the equatorial plane. To this end, 15 radial lines were randomly drawn on the image of the equatorial plane and fluorescence at each pixel was recorded.

#### 2.5.7. Statistical Analysis

Statistical analysis of all biological data was performed by means of one- or two-way ANOVA, with Bonferroni’s test for multiple comparisons, using GraphPad PRISM 4.03 software.

## 3. Results

### 3.1. Photodynamic Characteristics of BODIPYs 3 and 6

The photodynamic features of BODIPYs **3** and **6** in terms of lipophilicity and ^1^O_2_ generation capability were characterized. In particular, both BODIPYs had higher molar extinction coefficient values, compared to their bromine precursors [29], most likely due to the introduction of the pyridinium group favoring dissolution in DMSO (Table 1).

Despite the presence of the pyridinium group and the positive charge, the octanol/water partition coefficients (LogP, Table 1) seemed to indicate the higher solubility of **3** and **6** in the organic phase, as compared to the aqueous one. This behavior could be explained by the large neutral part of the molecules and the presence of the two iodine atoms. As expected, compound **6**, harboring a chain of eight carbon atoms, proved to be more lipophilic than **3** with its appendix of four carbon atoms.

The relative rates of ^1^O_2_ production of **3** and **6** were obtained by comparison with Rose Bengal (RB), known as the most powerful singlet oxygen producer [29]. Considering equal to 1 as the ^1^O_2_ production from RB, compounds **3** and **6** developed 0.8 and 0.6 ^1^O_2_, respectively, therefore accounting for good performances of the two BODIPYs.

### 3.2. Nanoparticle Synthesis and Characterization

**Figure 1:**
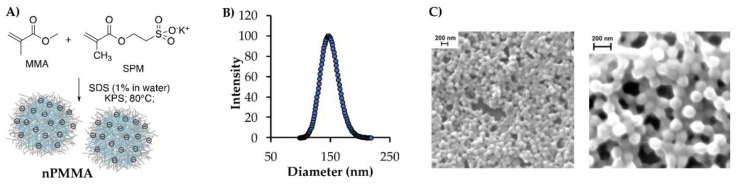
(**A**) Nanoparticle synthesis and schematic representation. (**B**) Representative example of nPMMA hydrodynamic diameter distribution. (**C**) The nPMMA SEM analysis (scale bar 200 nm) at two different magnifications, e.g., 42,000× and 125,000×.

Negatively charged core-shell polymethyl methacrylate nanoparticles (nPMMAs) were synthesized by an emulsion co-polymerization reaction (Figure 1A), affording nanoparticles with an average hydrodynamic diameter of 145 ± 5 nm (Figure 1B) and a zeta potential of −51.4 mV. The morphological analysis, performed by scanning electron microscopy (SEM), indicated that the particles were spherical in shape and regularly distributed, with an average dry diameter of 132 ± 4 nm. The difference in the NPs’ radii, observed with DLS and SEM techniques, could be ascribed to the different environments in which the measurements were performed, e.g., water and air, respectively (Figure 1C).

**Figure 2:**
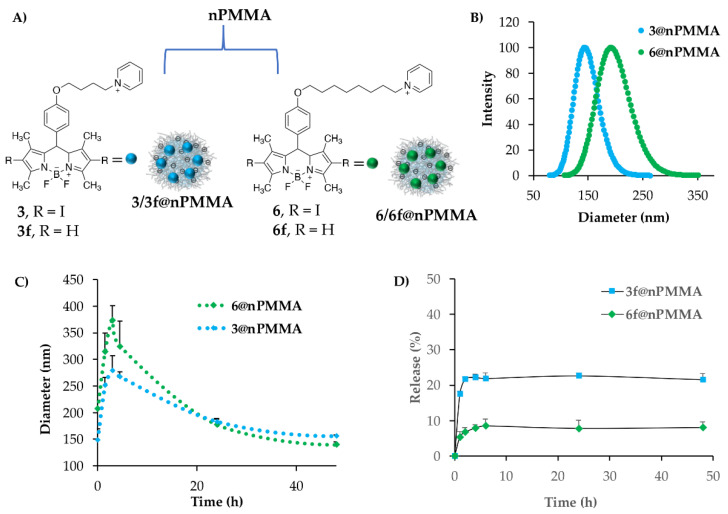
(**A**) Schematic representation of **3/3f@nPMMA** and **6/6f@nPMMA** nanoparticles. (**B**) Representative DLS measurements of **3@nPMMA** (blue circles) and **6@nPMMA** (green circles) nanoparticles. (**C**) Particle size stability of **3@nPMMA** (blue circles) and **6@nPMMA** (green circles) nanoparticles in FBS 20% in PBS pH 7.4, *v*/*v*, as determined by DLS analysis. (**D**) Release profiles of **3f** and **6f** from nPMMA nanoparticles in PBS pH 7.4, as determined by spectrophotometric analysis.

Optimization experiments performed by fine-tuning of the NPs/BODIPYs ratio, determined the best loading conditions in terms of efficiency (96%) and loading content, which resulted in 3.75% and 4.2% for **3@nPMMA** and **6@nPMMA**, respectively (Figure 2A). Under these conditions, we obtained stable and reproducible nanoparticles with an average hydrodynamic diameter of 144 ± 5 nm (PDI = 0.12) and 205 ± 11 nm (PDI = 0.2) for **3@nPMMA** and **6@nPMMA**, respectively (Figure 2B). The zeta potential values of nanoparticles after loading were −25.07 and −19.02 for **3@nPMMA** and **6@nPMMA**, respectively, indicating that the overall negative shell was preserved, and, thus, favoring their colloidal stability.

Indeed, the stability of BODIPY-loaded nPMMA nanoparticles was evaluated at 37 °C for 48 h in FBS 20% in PBS pH 7.4, *v*/*v* (Figure 2C). Our results indicated a similar stability trend for both nanoparticles: an initial diameter increase was observed during the first 4 h of the experiment, after which, the particles’ sizes gradually decreased until reaching the initial value, and then stayed stable for 15 days. Therefore, although the presence of 20% FBS induced an initial size increase, likely because of the interaction with serum proteins, the sizes of the nanoparticles remained within an acceptable range (<200 nm), and no visible aggregation/precipitation phenomena were observed.

Release experiments were performed by spectrophotometric analysis taking advantage of the fluorescent version of BODIPYs, namely **3f** and **6f** (Figures S1–S5), and were performed by the dialysis method, using, as release medium, PBS pH 7.4, owing to the solubility of both fluorescent BODIPYs in aqueous media. The BODIPYs’ release from the nanoparticles was lower than 10% for **6f@nPMMA** and around 20% of the loaded amount for **3f@nPMMA**, reaching a plateau after 6 h (Figure 2D). These data indicated that the electrostatic bond between the positively charged BODIPYs and nPMMA nanoparticles was satisfactorily stable for biological application. The lower release observed for **6f**, as compared to **3f**, could be ascribed to the longer chain of this BODIPY, favoring its hydrophobic interaction with the NP shell, and, thus, overall increasing the loading stability.

**Figure 3:**
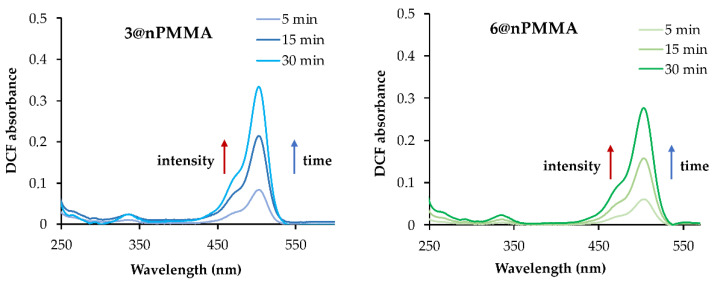
ROS production of **3@nPMMA** and **6@nPMMA** nanoparticles displayed as absorption spectra of DCF measured at different irradiation times (minutes).

The ROS generation of **3** and **6** loaded onto nPMMA nanoparticles was evaluated spectrophotometrically by measuring the increase in the absorption peak at 500 nm of 2,7-dichlorofluorescein (DCF). Indeed, DCF was only produced in the presence of ROS (see baseline Figure S6) upon subsequent hydrolysis and oxidation of the H_2_DCFDA probe (see Section 2.4.2) [30]. A solution of **3**/**6@nPMMA** nanoparticles and ROS probe was irradiated with a white tungsten lamp (300 W) for different time intervals and the DCF absorbance was recorded. The **3@nPMMA** and **6@nPMMA** displayed very similar ROS-producing abilities (Figure 3) and were comparable to those of free **3** and **6** (Figure S7). The absorbance peak at 500 nm increased with the irradiation time in a light dose-dependent manner.

### 3.3. Photodynamic Activity in 2D Cell Lines

#### 3.3.1. Effect on Cell Viability

The photodynamic activity of **3**, **6**, **3@nPMMA** and **6@nPMMA** on HCT116, SKOV3, MCF7 and MDA-MB231 cells was assessed by MTT assay upon treatment with increasing BODIPY concentrations, followed by light irradiation and 24 h incubation in a drug-free medium. The IC_50_ values were obtained from the corresponding dose–response curves (Table 2). All the tested compounds/formulations showed cytotoxic effects at nanomolar concentrations. In particular, in three out of four cell lines, **3@nPMMA** was as potent as the corresponding unbounded derivative **3**, while **6@nPMMA** was always significantly less potent than **6**. The intrinsic cytotoxicity of the photosensitizers was assessed by omitting the irradiation step from the treatment protocol and was found to be negligible.

#### 3.3.2. Cellular Uptake

To get insight on the different cytotoxic effects exerted by **3@nPMMA** and **6@nPMMA**, as compared to their free forms, we studied the cellular uptake behavior of our formulations. Indeed, it is widely acknowledged that the cellular uptake/localization of PSs and their ROS generation capability are crucial elements to the successful application of PDT, and that different cellular responses could be ascribed to diverse uptake behaviors [31]. 

**Figure 4:**
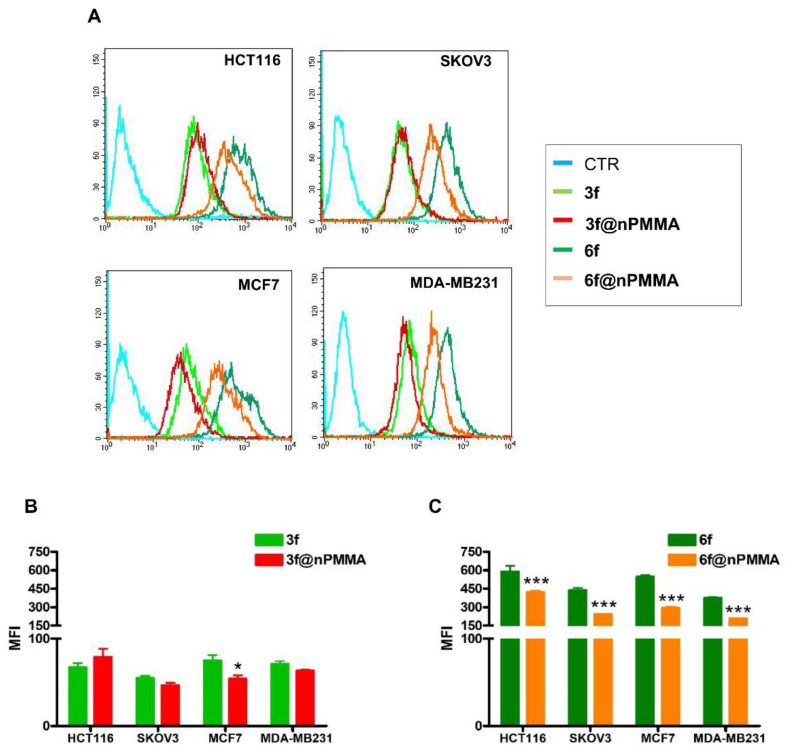
Uptake of **3f** (light green histograms or bars), **3f@nPMMA** (red histograms or bars), **6f** (dark green histograms or bars) and **6f@nPMMA** (orange histograms or bars) in monolayer cultured cell lines. The figure shows a representative flow cytometric analysis out of 3 independent experiments with similar results (**A**). The light blue histogram represents controls. Fluorescence intensity was quantitated based on the Median Fluorescence Intensity (MFI) and results obtained in 3 independent experiments are reported in the graphs (**B**) (**3**) and (**C**) (**6**) (mean ± SE; * *p* < 0.05 and *** *p* < 0.001 vs. free molecules).

In our study, cellular internalization was assessed by taking advantage of the fluorescent analogs of **3** and **6**, namely **3f** and **6f**, and their nanoparticles loaded version (**3f@nPMMA** and **6f@nPMMA**). Our data showed that upon 24 h incubation in the presence of 100 nM of **3f**, **6f**, **3f@nPMMA** and **6f@nPMMA**, both **6f** formulations exhibited higher intracellular accumulation, as compared to **3f** formulations (Figure 4A). The **3f** and **3f@nPMMA** showed a similar accumulation trend in HTC116, SKOV3 and MDA-MB231 cell lines, while in MCF7 cells **3f** internalization was significantly higher, in respect to **3f@nPMMA** (Figure 4B), and in good agreement with MTT results (Table 2). Furthermore, as compared to **6f@nPMMA**, free **6f** exhibited greater accumulation in all cell lines (Figure 4C), in accordance with the higher cytotoxicity of **6**, in respect to **6@nPMMA**.

#### 3.3.3. PDT-Induced Cell Death

**Figure 5:**
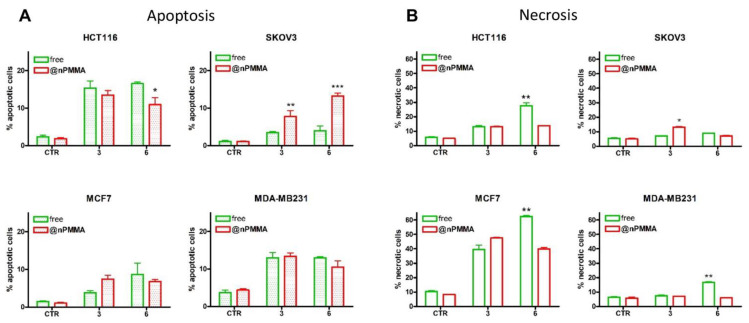
Percentage of apoptotic (**A**) and necrotic (**B**) HCT116, SKOV3, MCF7 and MDA-MB231 cells following 24 h treatment with free (green) or nPMMA-bounded (red) **3** and **6**, 1h irradiation and 24 h incubation in drug-free medium (mean ± SE of 3/4 independent experiments; * *p* < 0.05, ** *p* < 0.01 and *** *p* < 0.001 vs. respective free-BODIPY).

It has been largely reported that PDT triggers cell death, mainly through apoptosis and necrosis [32,33]. In agreement with this evidence, our results indicated that apoptosis played a major role in tumor cell death induced by the four compounds in three out of four cell lines. Different extents of apoptosis were observed when HCT116, SKOV3 and MDA-MB231 cells were treated with equitoxic concentrations of the compounds. As shown in Figure 5A, **3** and **3@nPMMA** induced the same extent of apoptotic cell death in HCT116 and MDA-MB231 cells, while in SKOV3 and MCF7 cells **3@nPMMA** induced a significantly higher percentage of apoptosis than **3**. Both **6** and **6@nPMMA** induced similar percentages of apoptosis in MCF7 and MDA-MB231 breast cancer cells, while a different trend was observed in SKOV3 and HCT116 cells, with **6@nPMMA** being more effective than **6** in SKOV3 cells.

The analysis of necrosis induction in our model cell lines showed that all formulations induced a low necrotic cell death in HCT116, SKOV3 and MDA-MB231 cells (Figure 5B), although a significant increase in necrotic cells, over controls, was observed in HCT116 and MDA-MB231 cells treated with **6** and in SKOV3 treated with **3@PMMA**. Interestingly, high levels of necrosis were observed in MCF7 cells, most probably compensating the lower extent of apoptotic cell death in this cell line. These results were in accordance with literature data, indicating that MCF7 was rather refractory to PDT-induced apoptosis, while being more likely to undergo necrosis [34,35]. These data indicated different cell type apoptotic/necrotic responses to equitoxic concentrations of free and nPMMA-bounded **3** and **6**.

#### 3.3.4. Effects on Cell Migration

**Figure 6:**
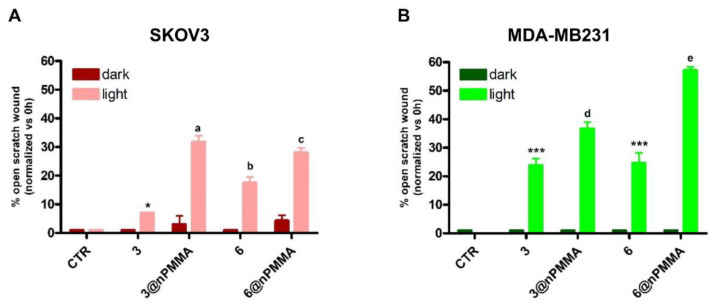
Percentage of open scratch wound, normalized vs. 0 h, in SKOV3 (**A**) and MDA-MB231 (**B**) cells following 24 h incubation with subtoxic concentrations of **3**, **3@nPMMA**, **6** and **6@nPMMA**, 1 h irradiation and incubation for 24 h in drug free medium at 37 °C (mean ± SD of three independent experiments; **a** *p* < 0.001 vs. CTR and **3@nPMMA** dark, *p* < 0.01 vs. **3** light; **b** *p* < 0.001 vs. CTR and **6** dark, *p* < 0.05 vs. **6@nPMMA**; **c** *p* < 0.001 vs. CTR and **6@nPMMA** dark, *p* < 0.05 vs. **6** light; * *p* < 0.05 vs. CTR and **3** dark; *** *p* < 0.001 vs. C and same treatment dark; **d** *p* < 0.001 vs. CTR and **3@nPMMA** dark, *p* < 0.01 vs. **3** light; **e** *p* < 0.001 vs. CTR and **6@nPMMA** dark, *p* < 0.001 vs. **6** light).

In a previous paper, we demonstrated that HCT116 and MCF7 cells did not show intrinsic migratory capacity under conditions similar to those used in the current study. Therefore, the effects of subtoxic concentrations (IC_20_) of **3**, **3@nPMMA**, **6**, and **6@nPMMA** on cell migration were assessed only on SKOV3 and MDA-MB231 cells, using the scratch wound healing assay. The scratch was monitored through a camera connected to a microscope and pictures were taken immediately after the treatment with the four formulations (t0) and after 24 h in the dark or upon irradiation. Importantly, using subtoxic compound concentrations avoided massive cell death, allowing evaluation of the effects of PDT on cell migration. The representative images obtained are shown in Figures S8 and S9. Furthermore, to better represent the scratch wound healing results, percentages of the open scratch wound detected at 24 h were normalized vs. the same percentage at t0 (Figure 6). In the absence of, or upon treatment with, nude nPMMA nanoparticles, both cell lines completely covered the surface during the 24 h observation time, regardless of the presence of the irradiation step (Figures S8 and S9).

In the absence of irradiation, no significant inhibition of cell migration over control was observed in both cell lines, indicating that photoactivation was a necessary condition to elicit the antimigratory effect of the compounds.

Following photoactivation, a significant inhibition of cell migration was observed in both cell lines for all tested compounds. Interestingly, the nPMMA-bounded BODIPYs were significantly more potent than the free molecules in reducing cell migration (Figure 6).

The balance between extracellular matrix destruction and deposition is essential for maintaining tissue architecture and functions. The degradation of the extracellular matrix, and of the basal membrane, is the first step for cancer cell migration and invasion. Matrix metalloproteinases (MMPs), and their tissue inhibitors, are known to modulate these processes, and higher expression of different MMPs facilitates tumor cell invasion and metastasis [36,37]. In human cancers, a strong correlation has been reported between high levels of MMPs and invasiveness [38]. In particular, MMP2 (gelatinase-A) and MMP9 (gelatinase-B) are deeply associated with the presence of metastatic tumors [39].

**Figure 7:**
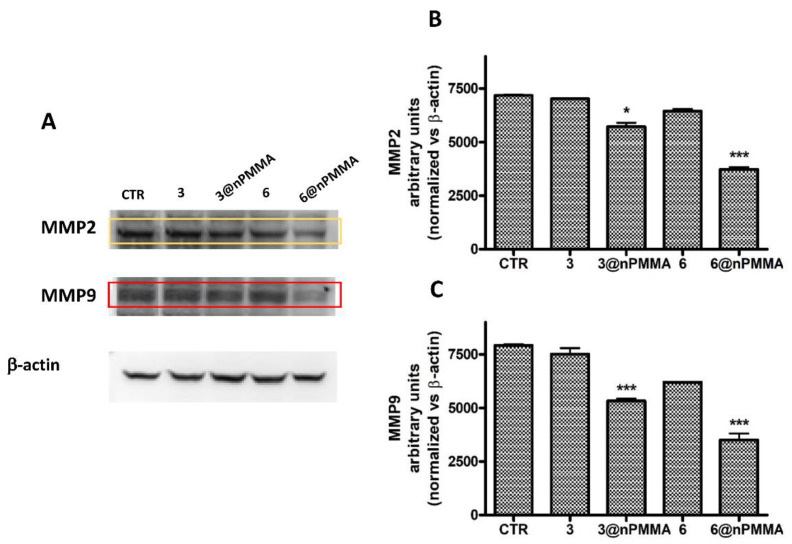
MMP2 and MMP9 protein levels and densitometric analysis in SKOV3 cells treated 24 h with subtoxic concentrations (IC_20_) of **3**, **3@nPMMA**, **6** and **6@nPMMA**, irradiated for 1 h and incubated for 24 h in a drug free medium at 37 °C. (**A**) Representative western blotting images for MMP2, MMP9 and β-actin protein levels. Densitometric analysis of MMP2 (**B**) and MMP9 (**C**) performed for all the experiments. The results were normalized vs. β-actin protein levels (* *p* < 0.05, *** *p* < 0.001 vs. CTR and free BODIPY).

**Figure 8:**
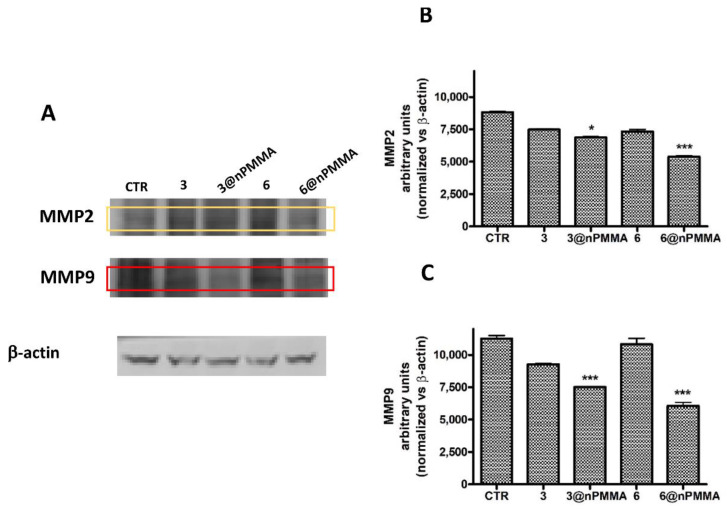
MMP2 and MMP9 protein levels and densitometric analysis in MDA-MB231 cells treated 24 h with subtoxic concentrations (IC_20_) of **3**, **3@nPMMA**, **6** and **6@nPMMA**, irradiated for 1 h and incubated for 24 h in a drug free medium at 37 °C. (**A**) Representative western blotting images for MMP2, MMP9 and β-actin protein levels. Densitometric analysis of MMP2 (**B**) and MMP9 (**C**) performed for all the experiments. The results were normalized vs. β-actin protein levels (* *p* < 0.05, *** *p* < 0.001 vs. CTR and free BODIPY).

To evaluate if the antimigratory effects observed in SKOV3 and MDA-MB231 cells, following treatment with **3**, **3@nPMMA**, **6** and **6@nPMMA,** could be related to their abilities to induce alterations in MMP2 and MMP9 expression, we analyzed their protein levels following PDT with the same subtoxic concentrations used for the scratch wound healing assay. Our results indicated that nPMMA-bounded BODIPYs could significantly reduce MMP2 and MMP9 protein levels in both cell lines, compared to free BODIPYs and controls, confirming their anti-migratory effect (Figure 7 and Figure 8).

### 3.4. Photodynamic Activity in 3D Spheroids

#### 3.4.1. Effects on Viability

**Figure 9:**
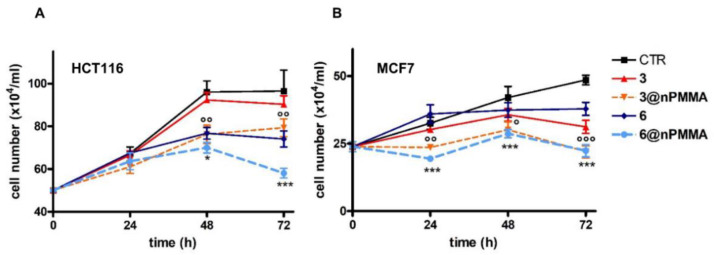
Growth curves of HCT116 (**A**) and MCF7 (**B**) spheroids following 24 h treatment with **3**, **3@nPMMA**, **6** and **6@nPMMA** at concentrations corresponding to the IC_50_ values obtained in monolayer-cultured cells, 1 h irradiation and 24 h incubation in a drug-free medium in the dark. Counts of viable cells were performed immediately following irradiation (time 0) and 24, 48 and 72 h later (mean ± SE of 3/5 spheroids; ° *p* < 0.05, °° *p* < 0.01, °°° *p* < 0.001 vs. 3; * *p* < 0.05, *** *p* < 0.001 vs. 6).

Previous studies from our group performed on HCT116, SKOV3, MCF7 and MDA-MB231 cell lines, indicated that only HCT116 and MCF7 cells could form spheroids [40]. Therefore, the photodynamic effects of **3**, **3@nPMMA**, **6** and **6@nPMMA** on 3D cultured cells were only evaluated on spheroids obtained from these two cell lines, and Figure 9 shows the obtained growth curves.

Control spheroids from both HCT116 and MCF7 cells increased their size following 24, 48 and 72 h incubation, as indicated by the increasing cell number. In particular, the HCT116 spheroids experienced faster growth than the MCF7 spheroids. Treatment with all compounds, followed by photoactivation, induced a reduction in viable cell numbers in both HCT116 and MCF7 spheroids. Interestingly, **3@nPMMA** and **6@nPMMA** were significantly more potent than **3** and **6**. The intrinsic cytotoxicity of the BODIPYs was assessed by omitting the irradiation step from the treatment protocol and was found to be negligible in all cases.

#### 3.4.2. BODIPY Distribution and Intensity

**Figure 10:**
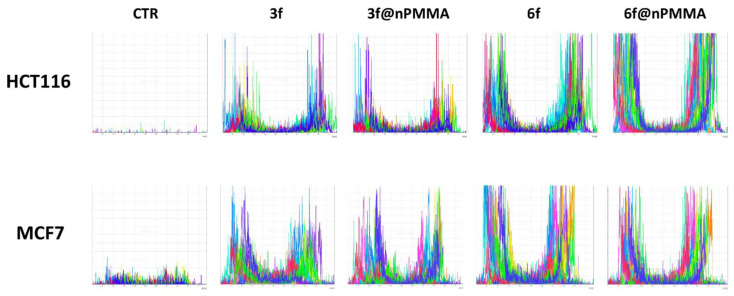
Penetration of 3f, 3f@nPMMA, 6f and 6f@nPMMA (100 nM) in HCT116 and MCF7 spheroids after 24 h incubation. The histograms represent the analysis of distribution and intensity of fluorescence in the PSs in 15 different randomly traced diameters in the equatorial planes.

Confocal microscopy analysis of the distribution and intensity of the fluorescence of the PSs in the images of the equatorial planes obtained on HCT116 and MCF7 spheroids showed that the penetration of fluorescent **3f** and **6f** compounds, free or loaded onto nPMMA nanoparticles, was limited to the external cell rim of the spheroids, while no appreciable penetration was detected in the inner core (Figure 10).

The internalization extent of compound **6f** was generally greater than **3f**. At the same time, no significant difference was observed in the fluorescence intensity of **3f@nPMMA** and **6f@nPMMA** in both cell lines compared to the free BODIPYs.

**Figure 11:**
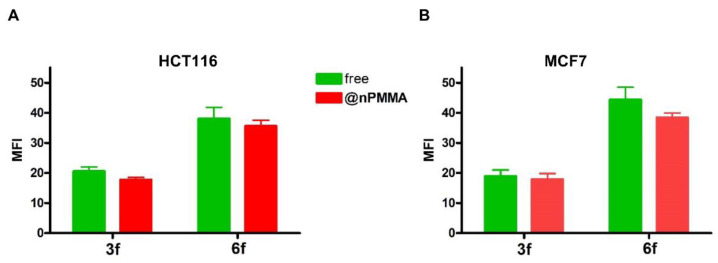
Uptake of **3f**, **3f@PMMA**, **6f** and **6f@PMMA** in cells from HCT116 (**A**) and MCF7 (**B**) spheroids (mean ± SE of 3 independent experiments; vs. free BODIPYs).

In agreement with confocal microscopy results, lower amounts of **3f**, compared with **6f,** were found in cells obtained from both HCT116 and MCF7 spheroids, following 24 h treatment with the free and nPMMA-bounded BODIPYs (100 nM). Furthermore, no differences in the amount of **3f@nPMMA** and **6f@nPMMA**, compared to free compounds, were observed in both cell lines (Figure 11).

## 4. Discussion

Although several photosensitizers are available for PDT application, some critical drawbacks, such as dark toxicity, poor molar extinction coefficients, low photostability, selectivity, and biocompatibility, limit their translation into clinical practice [32,41]. Furthermore, most clinically approved PSs are cyclic tetrapyrroles, whose purification, synthesis, or synthetic modification is challenging and low-yielding [42]. Finally, due to the lack of tumor selectivity, the therapeutically active PS dosage is relatively high, thus posing additional issues of unwanted phototoxicity and side effects [32].

Recent studies have shown that the BODIPY (4,4-difluoro-4-bora-3a,4a-diaza-s-indacene) scaffold represents an interesting chemical structure to develop PSs with improved features for clinical application, including PDT [9,10,11,12]. In addition, the use of suitably designed nanosystems for delivering PS molecules has been shown to be a promising approach in different preclinical anticancer PDT experiments [43,44,45]. In principle, loading the PS onto nanoparticles could favor its preferential accumulation at the target tissue, thanks to passive targeting, and protect the PS from degradation, thus, lowering the dosages and reducing the side effects.

In this work, we described, for the first time, the electrostatic loading of positively charged BODIPYs, namely **3** and **6**, on negatively charged PMMA nanoparticles, affording reproducible nanoparticles, e.g., **3@nPMMA** and **6@nPMMA**, with diameters in the 140–200 nm range and with high colloidal stability. With respect to positively charged PMMA nanoparticles, previously described by our group [22,23], nPMMA displayed lower intrinsic toxicity, while maintaining suitable internalization capacity (Figure 4, Figure 10 and Figure 11) and ROS generation ability (Figure 3).

From the biological point of view, **3@nPMMA** and **6@nPMMA** exhibited intriguing photodynamic effects in both 2D- and 3D-cultured cancer cell lines. In monolayer cells, **3@nPMMA** affected HCT116, SKOV3 and MDA-MB231 cellular viability at a similar extent to the free BODIPYs. On the other hand, a lower potency of **6@nPMMA** compared to **6** was observed in all 2D-cultured cell lines. Internalization experiments, performed by taking advantage of the fluorescent BODIPY versions, **3f** and **6f**, partially supported the observed differences in phototoxic behaviors (Figure 4), confirming the close relationship between PSs accumulation and phototoxic effect [21,31,46].

The cellular response to PDT is closely associated with the type of photochemical reaction induced by the PS, e.g., type I or type II, and depends on the PS photophysical/photochemical nature, its subcellular localization, and the oxygen concentration. Type I sensitizers lead to the formation of superoxide and hydroxyl radicals. Type II sensitizers mainly generate singlet oxygen (^1^O_2_) [47]. BODIPYs are generally acknowledged as type II sensitizers. Indeed, compounds **3** and **6** confirmed their ability to produce ^1^O_2_ (Table 1) and ROS both when free and when loaded onto nPMMA (Figure 3 and Figure S7). Unfortunately, under our experimental conditions, both free and nPMMA-bound BODIPYs did not show a significant ROS production in the four tested cell lines (Figure S10), probably due to the experimental settings.

It is generally recognized that, through the production of ROS and/or ^1^O_2_, type I and type II photochemical reactions may trigger one or various cell death mechanisms, mainly apoptosis, necrosis and autophagy, that directly affect cancer cells and lead to tumor destruction [33,48,49]. Our results indicated that treatment with equitoxic concentrations of **3**, **3@nPMMA**, **6** and **6@nPMMA** resulted in a different cell-type apoptotic response. In particular, the similar, or better, pro-apoptotic effects of the nPMMA-bound BODIPYs, compared to the free molecules, indicated a photodynamic advantage of the nPMMA formulations over free BODIPYs.

A key feature of an effective PDT treatment is the ability to accumulate and penetrate the inner and hypoxic core of the tumor mass. Indeed, the lower oxygen content and the non-homogeneous PS distribution would lead to reduced ROS generation and inefficient cell death. In this view, the use of 3D-cellular systems represents an increasingly considered model for mimicking the in vivo scenario, allowing gathering of important information on the potential pre-clinical transability of newly designed PDT systems [50,51]. Importantly, it was shown that within spheroids normoxic and hypoxic areas coexist, which make spheroids particularly useful for the study of new PSs [52].

Notably, in spheroids from HCT116 and MCF7 cells, **3@nPMMA** and **6@nPMMA** nanoparticles were significantly more potent than the free molecules (Figure 9). Considering the comparable internalization degree of the formulations in the HCT116 and MCF7 3D models, the observed higher cytotoxic effect exerted by **3@nPMMA** and **6@nPMMA** suggested better in vivo performances of the nPMMA-bound BODIPYs.

Finally, the greater inhibition of cell migration observed, following the PDT treatment with **3@nPMMA** and **6@nPMMA** on MDA-MB231 and SKOV3 (Figure 6), whose high motility correlates with a greater degree of malignancy [53], could be ascribed to MMP2 and MMP9 inhibition (Figure 7 and Figure 8). In fact, it is well known that the migratory behavior in tumor cells of epithelial origin is strongly related to the epithelial-to-mesenchymal transition (EMT) and is a harbinger of invasion and metastasis. A number of PSs have been reported to exhibit anti-migratory activity [53,54,55]. In addition, it has been reported that inhibition of EMT following PDT may occur through the inhibition of MMP2 and MMP9 [55,56] and account for the suppression of metastasis, thus ameliorating patient prognosis.

## 5. Conclusions

In conclusion, we reported the successful synthesis and characterization of negatively charged poly-methyl methacrylate nanoparticles as suitable and not intrinsically toxic carriers of positively charged BODIPYs. These novel nanosystems displayed effective PDT-mediated anticancer activity on a panel of solid tumor cell lines cultured both as 2D- and 3D-models. Most interestingly, compared to free molecules, BODIPY-bound PMMA nanoparticles were more effective in reducing cell viability in spheroids models, which better recapitulate the in vivo avascular tumor scenario. In addition, cell migration was significantly impaired upon BODIPY-bound nPMMA PDT treatment, and the inhibition of MMP2 and MMP9 was identified as a potential mechanism of the anti-migratory effect. These results, more evident on MDA-MB231 cells, which are widely recognized as less responsive to standard treatments and more prone to metastasize, support further investigations to validate this system into metastatic in vivo models of breast cancer. Finding alternative approaches to treat non-responsive primary tumors, while avoiding the spread of tumor cells to other sites, is crucial to increase the survival rate of patients and the quality of life experienced by them.

## Figures and Tables

**Table 1:** Wavelength of maximum absorption and extinction coefficient, partition coefficient octanol/water and ^1^O_2_ generation for BODIPYs **3** and **6**.

BODIPY	ε (M^−1^cm^−1^) ^a^	LogP	^1^O_2_ Generation ^b^
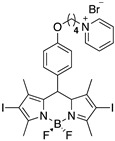 **3**	73,900 (534 nm)	0.44	0.8
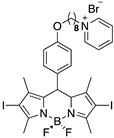 **6**	60,500 (534 nm)	1.23	0.6

^a^ DMSO was used as solvent; ^b^ Determined in isopropanol. Data were normalized vs. DPBF decay rate in the presence of Rose Bengal.

**Table 2:** IC_50_ values (nM) obtained in HCT116, SKOV3, MCF7 and MDA-MB231 cells by the MTT assay following 24 h incubation with the free and nPMMA-bounded BODIPYs (**3** and **6**), 1 h irradiation and 24 h incubation in a drug-free medium (mean ± SD of 4/5 independent experiments; * *p* < 0.05, ** *p* < 0.01 and *** *p* < 0.001 vs. respective free BODIPY).

Cell Line	3	3@nPMMA	6	6@nPMMA
HCT116	4.19 ± 0.53	3.16 ± 0.75	1.23 ± 0.31	5.65 ± 1.13 **
SKOV3	2.38 ± 0.37	4.27 ± 0.52	1.05 ± 0.14	12.72 ± 1.82 ***
MCF7	3.81 ± 1.03	7.47 ± 0.67 *	1.71 ± 0.46	16.77 ± 2.08 ***
MDA-MB231	2.54 ± 0.10	2.44 ±0.17	0.59 ± 0.09	8.09 ± 1.33 ***

## Data Availability

The data presented in this study are available in this article (and Supplementary Material).

## Supplementary Materials

The following supporting information can be downloaded at: https://www.mdpi.com/article/10.3390/cancers15010092/s1, Figure S1. (A) Chemical synthesis of compounds **3f** and **6f** starting from derivatives 1a and 1b. (B) Absorbance and emission spectra of compound **3f** and **6f**. Figure S2. 1HNMR of compound **3f**, (400 MHz, DMSO-d6). Figure S3. 13CNMR of compound **3f**, (100 MHz, DMSO-d6). Figure S4. 1HNMR of compound **6f**, (400 MHz, DMSO-d6). Figure S5. 13CNMR of compound **6f**, (100 MHz, DMSO-d6). Figure S6. Graph showing the baseline profile of dichlorofluorescein (DCF) at different irradiation times. Figure S7. Graphs showing the ROS production of **3** and **6** displayed as absorption spectra of DCF, measured at different irradiation times (minutes). Figure S8. Effect of 24 h treatment with **3**, **3@nPMMA**, **6**, and **6@nPMMA** and nPMMA, 1 h irradiation and incubation, for 24 h in a drug free medium at 37 °C, on the migratory activity of SKOV3 cells. Scratch wounds were produced before the irradiation step and pictures were taken immediately after it (0) and after 24 h, through a camera connected to an Olympus IX81 microscope. The pictures shown are representative of three independent experiments. Figure S9. Effect of 24 h treatment with **3**, **3@nPMMA**, **6**, and **6@nPMMA** and nPMMA, 1h irradiation and incubation for 24 h in a drug free medium at 37 °C, on the migratory activity of MDA-MB231 cells. Scratch wounds were produced before the irradiation step and pictures were taken immediately after it (0) and after 24 h, through a camera connected to an Olympus IX81 microscope. The pictures shown are representative of three independent experiments. Figure S10. ROS levels in HCT116, SKOV3, MCF7 and MDA-MB231 cells following 24 h treatment with free- (green) or nPMMA-bound (red) **3** and **6** and 1 h irradiation. Fluorescence intensity was quantitated, based on the Median Fluorescence Intensity (MFI), and the results obtained in three independent experiments are reported in the graph (* *p* < 0.05, ** *p* < 0.01 vs. respective free-BODIPY).
