# Supplementary material for: Free and Poly-Methyl-Methacrylate-Bounded BODIPYs: Photodynamic and Antimigratory Effects in 2D and 3D Cancer Models
Source: Cancers (Basel). 2022 Dec 23;15(1):92. doi: 10.3390/cancers15010092 (PMC9817850; doi:10.3390/cancers15010092)
Supplement: Supplementary file 1 [file cancers-15-00092-s001.zip › cancers-2096340-supplementary.pdf]

Free and Poly-Methyl-Methacrylate-Bounded BODIPYs: Photodynamic and Antimigratory Effects in 2D and 3D Cancer Models

Marco Ballestri, Emanuela Marras, Enrico Caruso, Fabrizio Bolognese, Miryam Chiara Malacarne, Elisa Martella, Matilde Tubertini, Marzia Bruna Gariboldi * and Greta Varchi *

Supporting Information

Table of Content

Figure S1. A) Chemical synthesis of compounds **3f** and **6f** starting from derivatives **1a** and **1b**. B) Absorbance and emission spectra of compound **3f** and **6f.** 2

Figure S2. ^{1}HNMR of compound **3f**, (400 MHz, DMSO-d6). 2

Figure S3. ^{13}CNMR of compound **3f**, (100 MHz, DMSO-d6). 3

Figure S4. ^{1}HNMR of compound **6f**, (400 MHz, DMSO-d6). 3

Figure S5. ^{13}CNMR of compound **6f**, (100 MHz, DMSO-d6) 4

Figure S6. Graph showing the baseline profile of the dichlorofluorescein (DCF) at different irradiation times. 4

Figure S7. Graphs showing the ROS production of 3 and 6 displayed as absorption spectra of DCF measured at different irradiation times (minutes). 5

Figure S8. Effect of 24h treatment with **3**, **3@nPMMA**, **6**, and **6@nPMMA** and nPMMA, 1h irradiation and incubation for 24 h in drug free medium at 37 °C on the migratory activity of SKOV3 cells. Scratch wounds were produced before the irradiation step and pictures were taken immediately after it (0) and after 24 h, through a camera connected to an Olympus IX81 microscope. Picture showed are representative of three independent experiments. 5

Figure S9. Effect of 24h treatment with **3**, **3@nPMMA**, **6**, and **6@nPMMA** and nPMMA, 1h irradiation and incubation for 24 h in drug free medium at 37 °C on the migratory activity of MDA-MB231 cells. Scratch wounds were produced before the irradiation step and pictures were taken immediately after it (0) and after 24 h, through a camera connected to an Olympus IX81 microscope. Picture showed are representative of three independent experiments. 6

Figure S10. ROS levels in HCT116, SKOV3, MCF7 and MDA-MB231 cells following 24h treatment with free (green) or nPMMA-bounded (red) **3** and **6** and 1h irradiation. Fluorescence intensity was quantitated based on the Median Fluorescence Intensity (MFI) and results obtained in 3 independent experiments are reported in the graph (*p<0.05, **p<0.01 vs respective free-BODIPY). 7

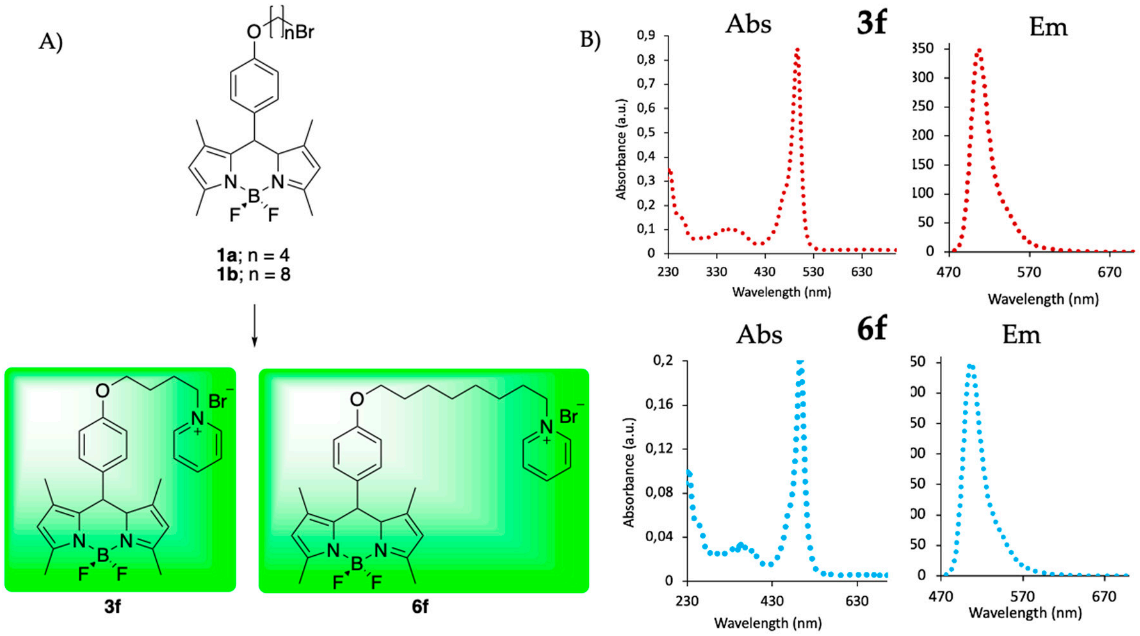

Figure S1. A) Chemical synthesis of compounds **3f** and **6f** starting from derivatives **1a** and **1b**. B) Absorbance and emission spectra of compound **3f** and **6f.**

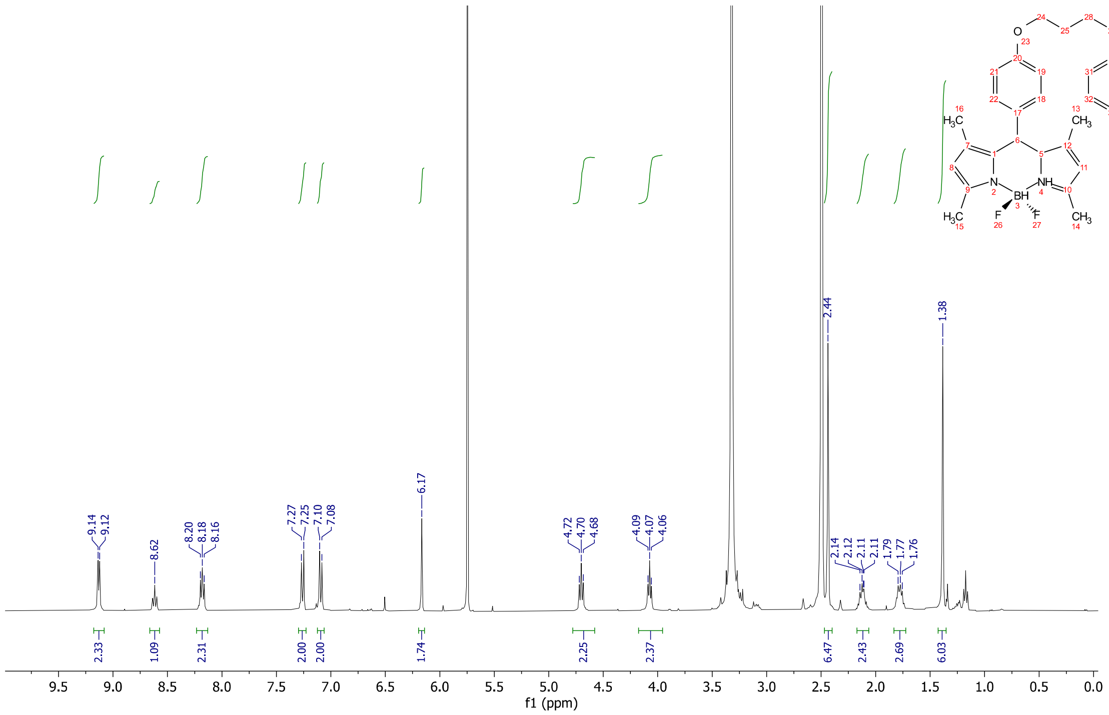

Figure S2. *^{1}HNMR of compound **3f**, (400 MHz, DMSO-d6).*

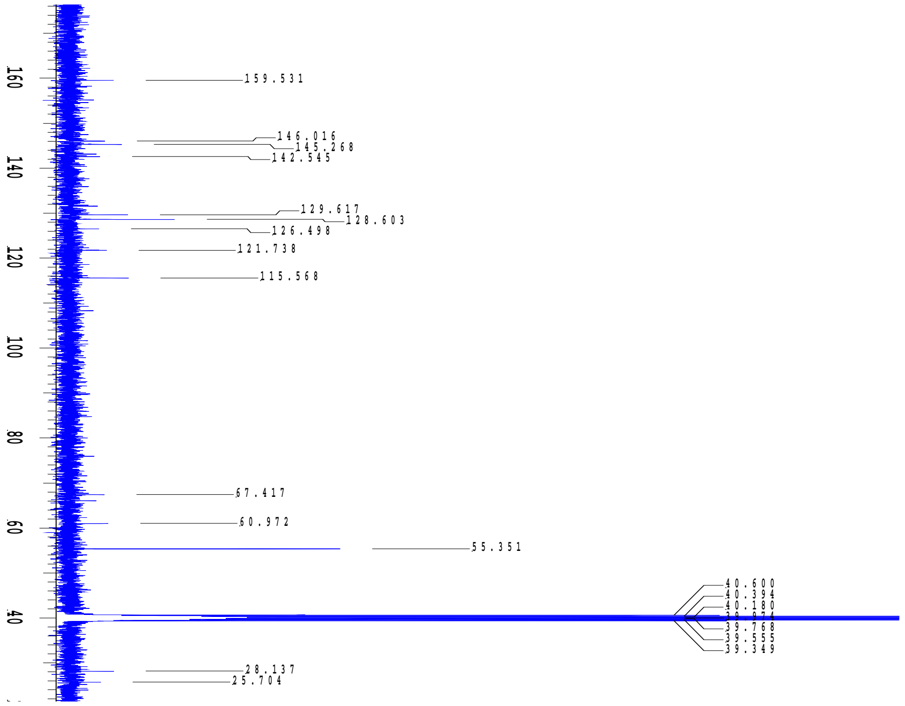

Figure S3. *^{13}CNMR of compound **3f**, (100 MHz, DMSO-d6).*

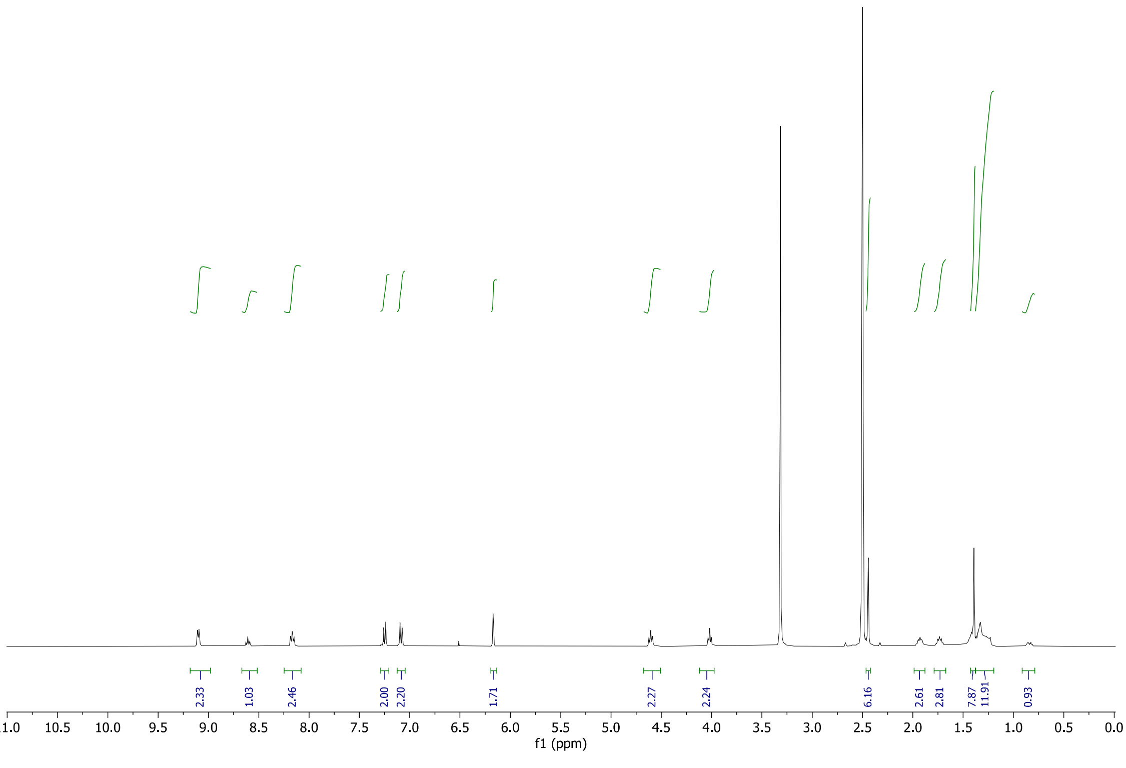

Figure S4. *^{1}HNMR of compound **6f**, (400 MHz, DMSO-d6).*

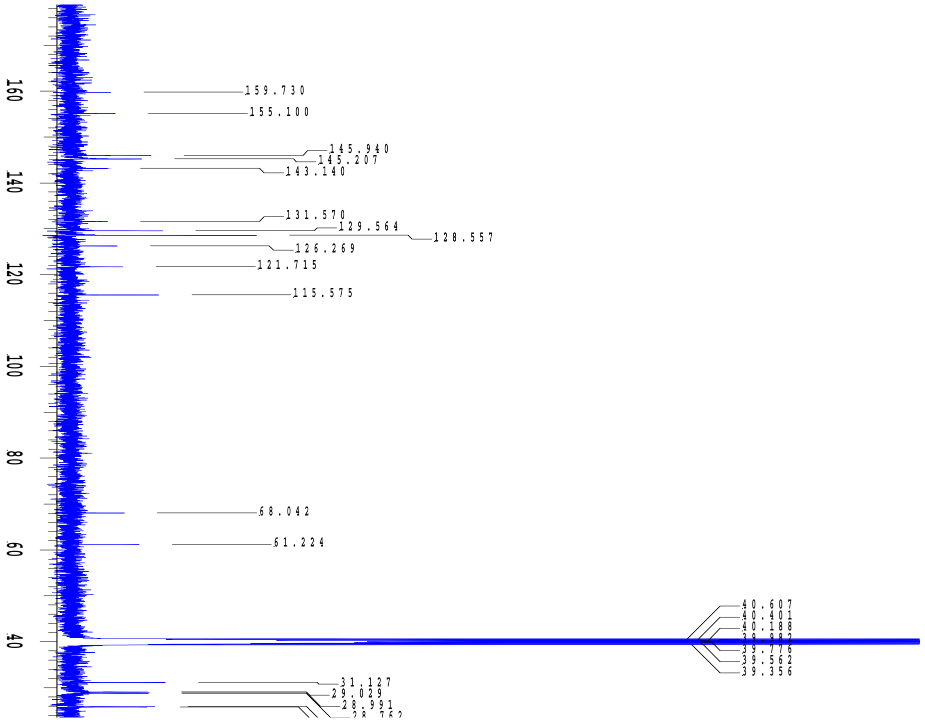

Figure S5. *^{13}CNMR of compound* ***6f****, (100 MHz, DMSO-d6)*

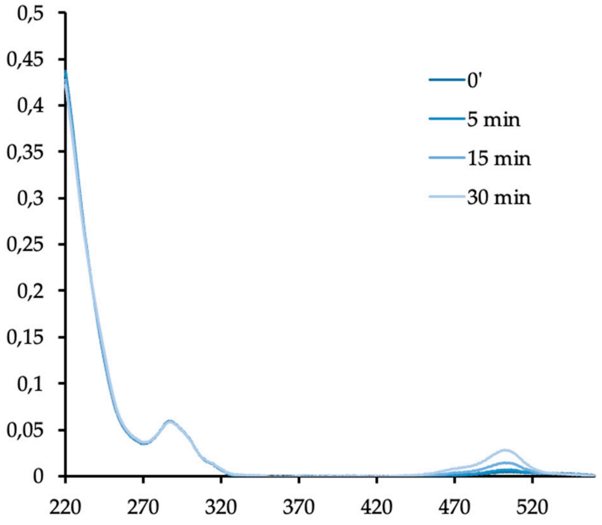

Figure S6. *Graph showing the baseline profile of the dichlorofluorescein (DCF) at different irradation times.*

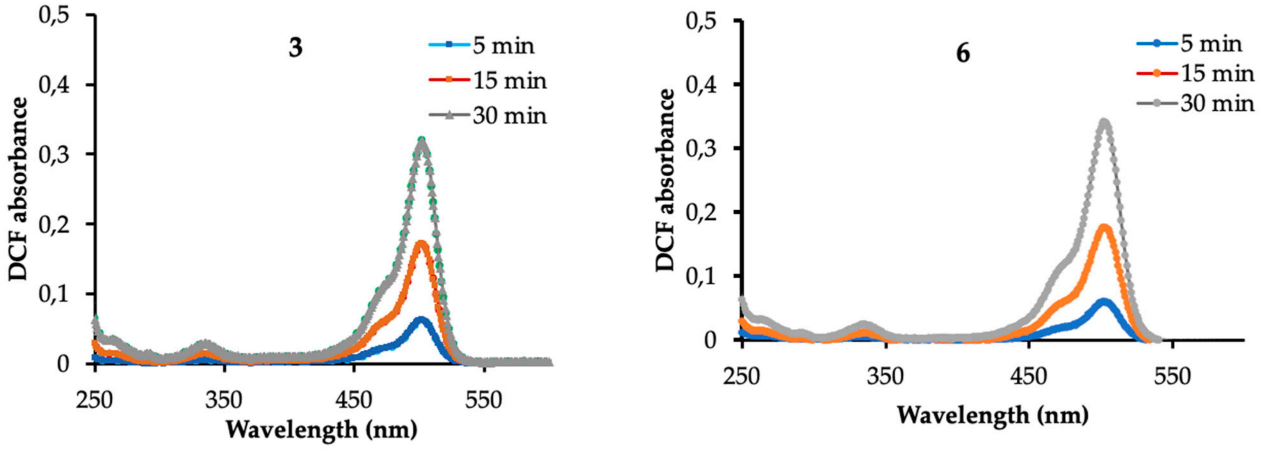

Figure S7. *Graphs showing the ROS production of 3 and 6 displayed as absorption spectra of DCF measured at different irradiation times (minutes).*

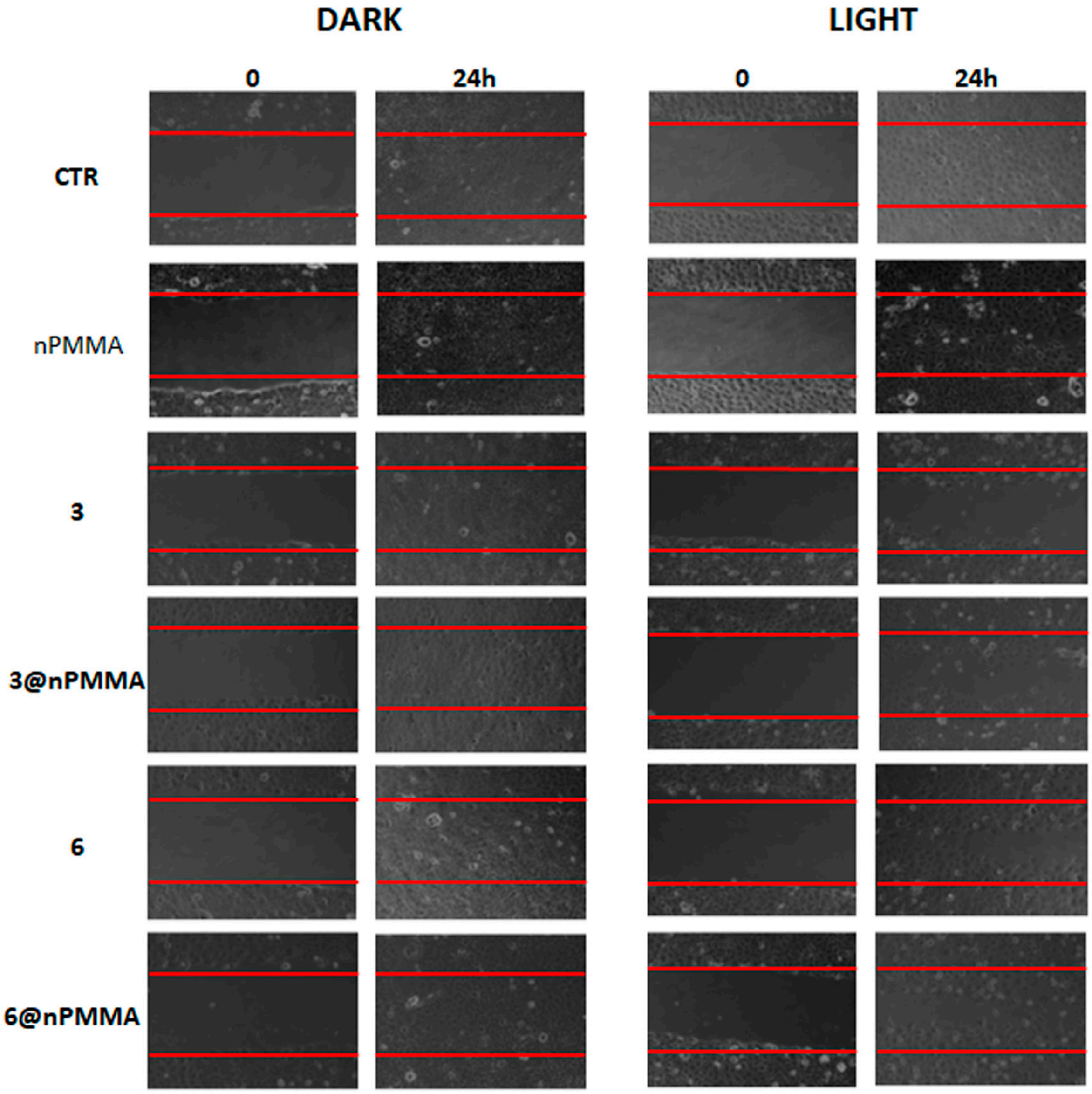

Figure S8. *Effect of 24h treatment with* ***3***, ***3@nPMMA***, ***6***, *and* ***6@nPMMA*** *and nPMMA, 1h irradiation and incubation for 24 h in drug free medium at 37 °C on the migratory activity of SKOV3 cells. Scratch wounds were produced before the irradiation step and pictures were taken immediately after it (0) and after 24 h, through a camera connected to an Olympus IX81 microscope. Picture showed are representative of three independent experiments*.

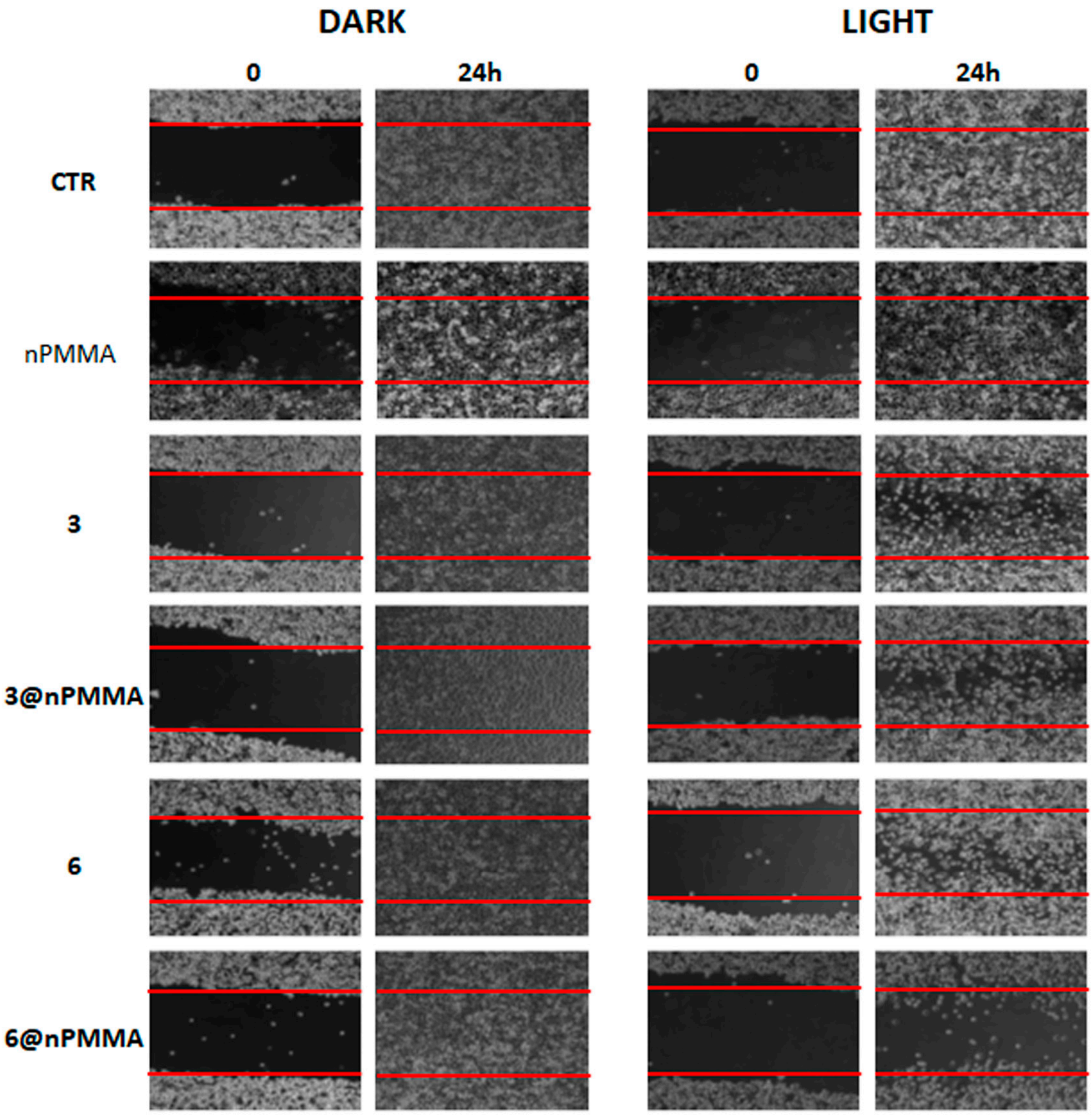

Figure S9. *Effect of 24h treatment with* ***3***, ***3@nPMMA***, ***6***, *and* ***6@nPMMA*** *and nPMMA, 1h irradiation and incubation for 24 h in drug free medium at 37 °C on the migratory activity of MDA-MB231 cells. Scratch wounds were produced before the irradiation step and pictures were taken immediately after it (0) and after 24 h, through a camera connected to an Olympus IX81 microscope. Picture showed are representative of three independent experiments.*

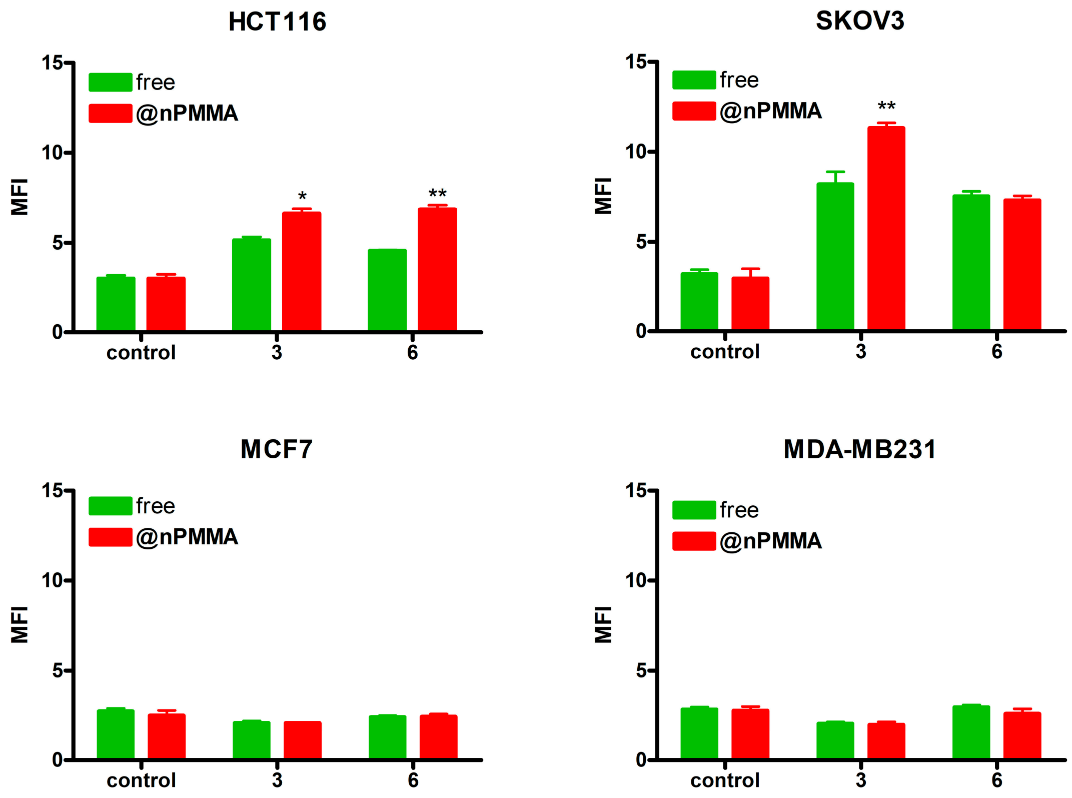

Figure S10. *ROS levels in HCT116, SKOV3, MCF7 and MDA-MB231 cells following 24h treatment with free (green) or nPMMA-bounded (red)* ***3*** *and* ***6*** *and 1h irradiation. Fluorescence intensity was quantitated based on the Median Fluorescence Intensity (MFI) and results obtained in 3 independent experiments are reported in the graph (*p<0.05, **p<0.01 vs respective free-BODIPY).*
